# Personal News Items

**Published:** 1916-05

**Authors:** 


### Personals.

Dr. and Mrs. A. H. Alsup, of Little River, are visiting in New Orleans.

Dr. Jim Watson has returned to Cameron after a long visit in Alabama.

Dr. H. M. Lanham, of Waco, is in Chicago taking a post-graduate course.

Dr. I. C. Munger, of Cozad, is in New York City taking a post-graduate course.

Dr. R. R. Allen, of Roby, is in New Orleans taking a post-graduate course in medicine.

Dr. George Blackwell, of Gorman, has gone to Chicago to take a post-graduate course.

Dr. Fred Hudson, of Jewett, is in New Orleans taking a post-graduate course in medicine.

Dr. and Mrs. S. R. Griffin, of Canyon, are on a several weeks visit to Galveston and New Orleans.

Dr. F. E. Rushing, of Fort Worth, has returned from a three months' post-graduate course in New York City.

Dr. J. W. Greenwood, of Memphis, has been elected department physician and surgeon by the fire department.

Dr. V. Gist, of Burkburnett, who has been confined to his room for some time with rheumatism is able to be out again.

Dr. S. A. Street and wife, of Wellington, have gone to Chicago, where the doctor will take a post-graduate course.

Dr. S. T. Humphreys is in Chicago attending a six weeks' course of post-graduate lectures at the Chicago Polyclinic.

Dr. S. W. Anthony and wife, of Dumas, are home again after spending the winter with relatives and friends in South Texas.

Dr. Devine, of Tom Bean, has been suffering from injuries sustained to his back when his buggy turned over with him recently.

Dr. L. W. Hollis, Jr., of Abilene, will be Assistat State Health Officer at the port of Galveston, succeeding Dr. Cook, who goes to Mexico.

Dr. Jennings-Wagner, of Palacios, has returned from Houston, where she has been for several weeks undergoing and recovering from an operation.

Dr. R. J. Blackwell, a prominent physician and druggist of Hawley, has been seriously ill recently, due to a rupture of a blood-vessel in the head.

Dr. W. H. O'Banion, Dr. and Mrs. D. B. Williamson, of Mendoza, and Dr. W. M. Pitts, of Luling, are in New Orleans taking a course in the Medical Department of Tulane University.

Dr. E. W. Loomis, city health officer of Dallas, left recently for New York, Boston and other Eastern cities, where he will study health problems for several weeks.

Dr. Ella Ware, of Stockdale, is able to resume her practice after a siege of erysipelas.

---

Gastritis.—There are probably few who are addicted to the use of alcohol who are entirely free from chronic gastritis. Tobacco is a conjoint cause.—*Ellingwood's.*

